# Supplementary material for: Fluoxetine Removal from Aqueous Solutions Using a Lignocellulosic Substrate Colonized by the White-Rot Fungus Pleurotus ostreatus
Source: Int J Environ Res Public Health. 2022 Feb 25;19(5):2672. doi: 10.3390/ijerph19052672 (PMC8910386; doi:10.3390/ijerph19052672)
Supplement: Supplementary file 1 [file ijerph-19-02672-s001.zip › ijerph-1590015-supplementary.pdf]

International Journal of
Environmental Research and Public Health

MDPI

SUPPLEMENTARY MATERIALS

Fluoxetine Removal from Aqueous Solutions Using a Lignocellulosic Substrate Colonized by the White-Rot Fungus *Pleurotus ostreatus*

A. D. M. Silva [1], J. Sousa [1], M. Hultberg [2], S. A. Figueiredo [1], O. M. Freitas [1,*] and C. Delerue-Matos [1]

[1] REQUIMTE/LAQV, Instituto Superior de Engenharia do Porto, Rua Dr. António Bernardino de Almeida 431, 4200-072 Porto, Portugal; andreia.silva@graq.isep.ipp.pt; 1191179@isep.ipp.pt; saf@isep.ipp.pt; cmm@isep.ipp.pt

[2] Department of Biosystems and Technology, Swedish University of Agricultural Sciences, P.O. Box 103, SE 230 53 Alnarp, Sweden; Malin.Hultberg@slu.se

* Correspondence: omf@isep.ipp.pt

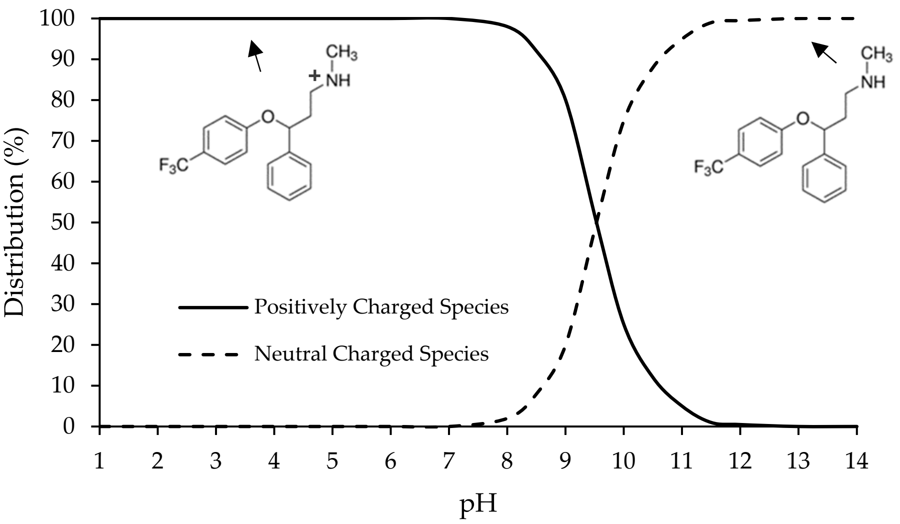

Figure S1. Species distribution diagram of fluoxetine as a function of pH (adapted from [2] and [3]).

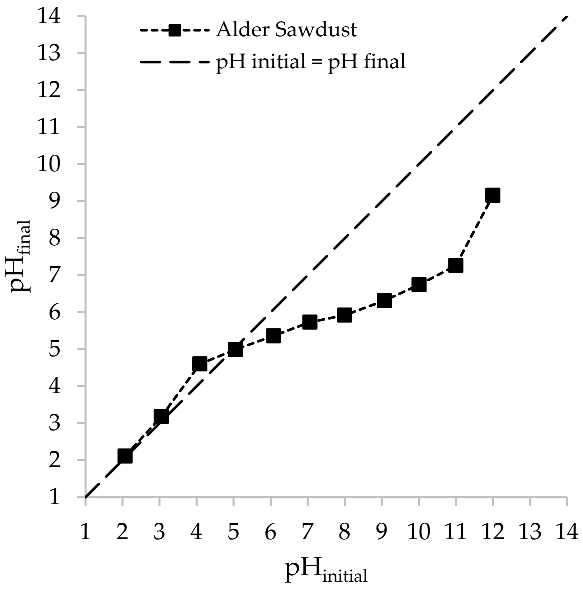

Figure S2. The point of zero charge (pH_{PZC}) determination in alder sawdust.

Table S1. Physicochemical properties of fluoxetine hydrochloride.

Molecular formula [(1)]	M.w. (g mol^{-1})	S.w. (mg L^{-1})	*p*Ka [(1)]	Log K_{ow} [(1)]
$C_{17}H_{18}F_3NO \cdot HCl$	345.79	4000	9.8	4.17

M.w.: Molecular weight; S.w.: Solubility in water; Ka: Acid dissociation constant; K_{ow}: Octanol-water partition coefficient.

[(1)] Source [1].

Table S2. List of reagents used in the study.

Reagent	Supplier	Country
Potassium bromide (KBr, for IR spectroscopy)	PanReac	Spain
Potassium nitrate (KNO_3)	Sigma-Aldrich®	USA
Sodium hydroxide (NaOH, purity >99.0%)	Labkem	Spain
Hydrochloric acid (HCl, purity 37% wt)	Honeywell Fluka™	Austria
2.6-dimethoxyphenol (2.6-DMP, purity 99%)	Labkem	Spain
Sodium acetate anhydrous (CH_3COONa, purity ≥99%)	Sigma-Aldrich	USA
Acetic acid glacial (CH_3COOH, purity ≥99.8%)	Carlo Erba	Spain
1-aminobenzotriazole (1-ABT)	Sigma-Aldrich	USa
Ortho-boric acid (H_3BO_3, purity 100%)	VWR BDH® Chemicals	Belgium
Acetic acid glacial (CH_3COOH, purity 99.8%, density 1.0501-1.0521)	CARLO ERBA Reagents	France
Ortho-phosphoric acid (H_3PO_4, purity 85%, density 1.70 kg L^{-1})	PanReac	Spain
Acetonitrile (CH_3CN, purity 99.99%)	VWR BDH® Chemicals HPLC-super gradient	France
Formic acid (HCOOH, purity 99%)	CARLO ERBA Reagents	France

Methanol (CH_3OH, purity >99.9%)	VWR BDH® Chemicals HPLC-super gradient	Netherlands

Table S3. Band assignments of FT-IR spectrum of alder sawdust.

Band	Wavenumber (cm^{-1})	Band assignments [1]	References
A	3408 ± 0	ν(O-H)	[4-8]
B	2925 ± 0	$\nu_{as}(CH_2)$ and $\nu_s(CH_2)$	[4-7]
C	1650 ± 1	ν(C=O)	[4, 7-9]
D	1539 ± 3	δ(C=C)	[8]
E	1401 ± 25	$\delta(CH_2)$ and $\delta(CH_3)$	[4, 8]
F	1238 ± 1	ν(C-O)	[8]
G	1079 ± 52	ν(C-O)	[4, 8, 9]
H	668 ± 1	Bending vibration modes of aromatic compounds.	[8]
I	601 ± 1		

[1] ν: stretching; ν_s: symmetric stretching; ν_{as}: asymmetric stretching; δ: deformation.

Table S4. pH and temperature values recorded at the beginning and the end of batch assays.

Assay	pH		Temperature (°C)	
	Beginning	End	Beginning	End
CMS Assays (1st day)	6.83 ± 0.19	6.69 ± 0.01	22.0 ± 0.0	24.0 ± 0.0
CMS Assays (15th day)	7.01 ± 0.01	6.66 ± 0.03	20.0 ± 0.0	24.0 ± 0.0
Biosorption Assays	6.94 ± 0.01	6.46 ± 0.00	24.0 ± 0.0	25.0 ± 0.0
CYP450 Inhibition Assays	6.75 ± 0.02	6.74 ± 0.05	23.0 ± 0.0	24.0 ± 0.0
CEE Assays	6.68 ± 0.01	6.68 ± 0.01	24.5 ± 0.7	24.5 ± 0.7

References

1. ChemAxon. Marvin Software. Available online: https://chemaxon.com (accessed on 10 January 2019)
2. Schneider, J.D. pH effects on the sorption of fluoxetine and sulfamethoxazole by three powdered activated carbons, in Civil and Environmental Engineering. Master of Science, Utah State University, Logan, Utah, May 2018.
3. Beardall, J. The Fate of Pharmaceuticals and Personal Care Products in Conventional and Engineered On-Site Wastewater Drain Fields, in Civil and Environmental Engineering. Master of Science, Utah State University, Logan, Utah, May 2015.
4. Wahab, M.A.; Jellali, S.; Jedidi, N. Ammonium biosorption onto sawdust: FTIR analysis, kinetics and adsorption isotherms modeling. *Bioresour. Technol*. **2010**, *101*, 5070-5075, doi:10.1016/j.biortech.2010.01.121.
5. Bajpai, S.K.; Bajpai, M.; Rai, N. Sorptive removal of ciprofloxacin hydrochloride from simulated wastewater using sawdust: Kinetic study and effect of pH. *Water SA*. **2012**, *38*, doi:10.4314/wsa.v38i5.4.
6. Abdullah, R.H.; Oda, A.M.; Omran, A.R.; Mottaleb, A.S.; Mubarakah, T.M. Study of Adsorption Characteristics a Low-Cost Sawdust for the Removal of Direct Blue 85 Dye from Aqueous Solutions. *Indonesian Journal of Chemistry*. **2018**, *18*, doi:10.22146/ijc.31536.

7. Saha, T.K.; Bishwas, R.K.; Karmaker, S.; Islam, Z. Adsorption Characteristics of Allura Red AC onto Sawdust and Hexadecylpyridinium Bromide-Treated Sawdust in Aqueous Solution. *ACS Omega*. **2020**, *5*, 13358-13374, doi:10.1021/acsomega.0c01493.
8. Dirckx, O.; Trouy, M.-C.; Merlin, A.; Deglise, X. Modifications de la couleur du bois d'Abies grandis exposé à la lumière solaire. *Annales des Sciences Forestières*. **1992**, *49*, 425-447, doi:10.1051/forest:19920501.
9. Jaya, H.; Omar, M.F.; Akil, H.M.; Ahmad, Z.A.; Zulkepli, N.N. Effect of Alkaline Treatment on Sawdust Reinforced High Density Polyethylene Composite under Wide Strain Rate. *Materials Science Forum*. **2016**, *840*, 103-107, doi:10.4028/www.scientific.net/MSF.840.103.
